# Supplementary material for: Norwegian moose CWD induces clinical disease and neuroinvasion in gene-targeted mice expressing cervid S138N prion protein
Source: PLoS Pathog. 2024 Jul 1;20(7):e1012350. doi: 10.1371/journal.ppat.1012350 (PMC11244775; doi:10.1371/journal.ppat.1012350)
Supplement: S1 Text — Fig A. PrPres in brain homogenates of gene-targeted mice and various CWD isolates. PrPres-positive brain homogenates of gene-targeted mice as indicated inoculated i.c. with R-CA1, R-NO16, and M-NO3 were analyzed by western blot along with CWD isolates from North America (Elk-CWD2, WTD-Wisc-1, WTD-116AG, and R-CA1) and Norway (R-NO16, M-NO3, and H-NO1) digested with 50 μg/ml of PK using anti-PrP antibodies 12B2. R-NO16 and H-NO1 did not harbor detectable amounts of PrPres. Fig B. Seeding activity in PrPres-negative brains of Prnp.Cer.Wt mice inoculated with R-NO16 and M-NO3. Left column shows RT-QuIC results from individual Prnp.Cer.Wt mice inoculated i.c. (n = 2) and i.p. (n = 1) with R-NO16 that were negative for PrPres on western blotting (Fig 2). Right column shows RT-QuIC results from individual Prnp.Cer.Wt mice inoculated i.p. with M-NO3 (n = 2) that were negative for PrPres on western blotting (Fig 2). Samples were considered positive when a minimum of two out of four reactions crossed the threshold relative fluorescence unit (RFU), indicated by the violet line. Positive dilutions are highlighted in black rectangles. The threshold is the average RFU of all negative control reactions plus five times their standard deviation. Negative control was a non-inoculated Prnp.Cer.Wt mouse brain. The y-axis represents the RFU, and the x-axis represents time in hours (h). Fig C. Representative immunohistochemistry of brain and spleen sections from gene-targeted mice inoculated i.p. with the R-NO16 and M-NO3 isolates. Abnormal PrP deposits were detected in the frontal cortex, cerebellum, and spleen of the Prnp.Cer.Wt mouse inoculated with R-NO16 (row 3, highlighted with the black rectangle), but not M-NO3 (row 5). No abnormal PrP deposits were detected in Prnp.Cer.138NN mice tissues inoculated with both isolates (rows 2 and 4). No abnormal PrP deposits were detected in the hippocampus of all mice tested. Detection of abnormal PrP deposits was performed using the anti-PrP a [file ppat.1012350.s002.docx]

***PLoS Pathogens PPATHOGENS-D-23-01939 R1***

**Supplementary Information**

**Norwegian moose CWD induces clinical disease and neuroinvasion in gene-targeted mice expressing cervid S138N prion protein**

Maria Immaculata Arifin^1¶^, Samia Hannaoui^1¶^, Raychal Ashlyn Ng^1^, Doris Zeng^1^, Irina Zemlyankina^1^, Hanaa Ahmed-Hassan^1,2^, Hermann M. Schatzl^1,3,4^, Lech Kaczmarczyk^5^, Walker S. Jackson^5^, Sylvie L. Benestad^6^ and Sabine Gilch^1,3,4^*

**Author affiliations:**

^1^ Faculty of Veterinary Medicine, University of Calgary, Calgary, Canada

^2^ Zoonoses Department, Faculty of Veterinary Medicine, Cairo University, Giza 12211, Egypt

^3^ Hotchkiss Brain Institute, University of Calgary, Calgary, Canada

^4^ Snyder Institute for Chronic Diseases, University of Calgary, Calgary, Canada

^5^ Linköping University, Linköping, Sweden

^6^ Norwegian Veterinary Institute, Ås, Norway

^*^[sgilch@ucalgary.ca](mailto:sgilch@ucalgary.ca)

¶Contributed equally`

**
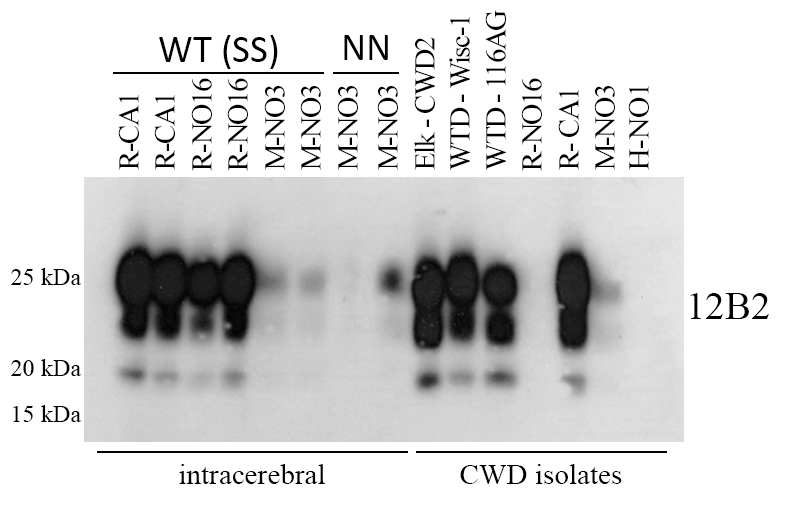
**

**Figure A. PrP^res^ in brain homogenates of gene-targeted mice and various CWD isolates.** PrP^res^-positive brain homogenates of gene-targeted mice as indicated inoculated i.c. with R-CA1, R-NO16, and M-NO3 were analyzed by western blot along with CWD isolates from North America (Elk-CWD2, WTD-Wisc-1, WTD-116AG, and R-CA1) and Norway (R-NO16, M-NO3, and H-NO1) digested with 50 μg/ml of PK using anti-PrP antibodies 12B2. R-NO16 and H-NO1 did not harbor detectable amounts of PrP^res^.


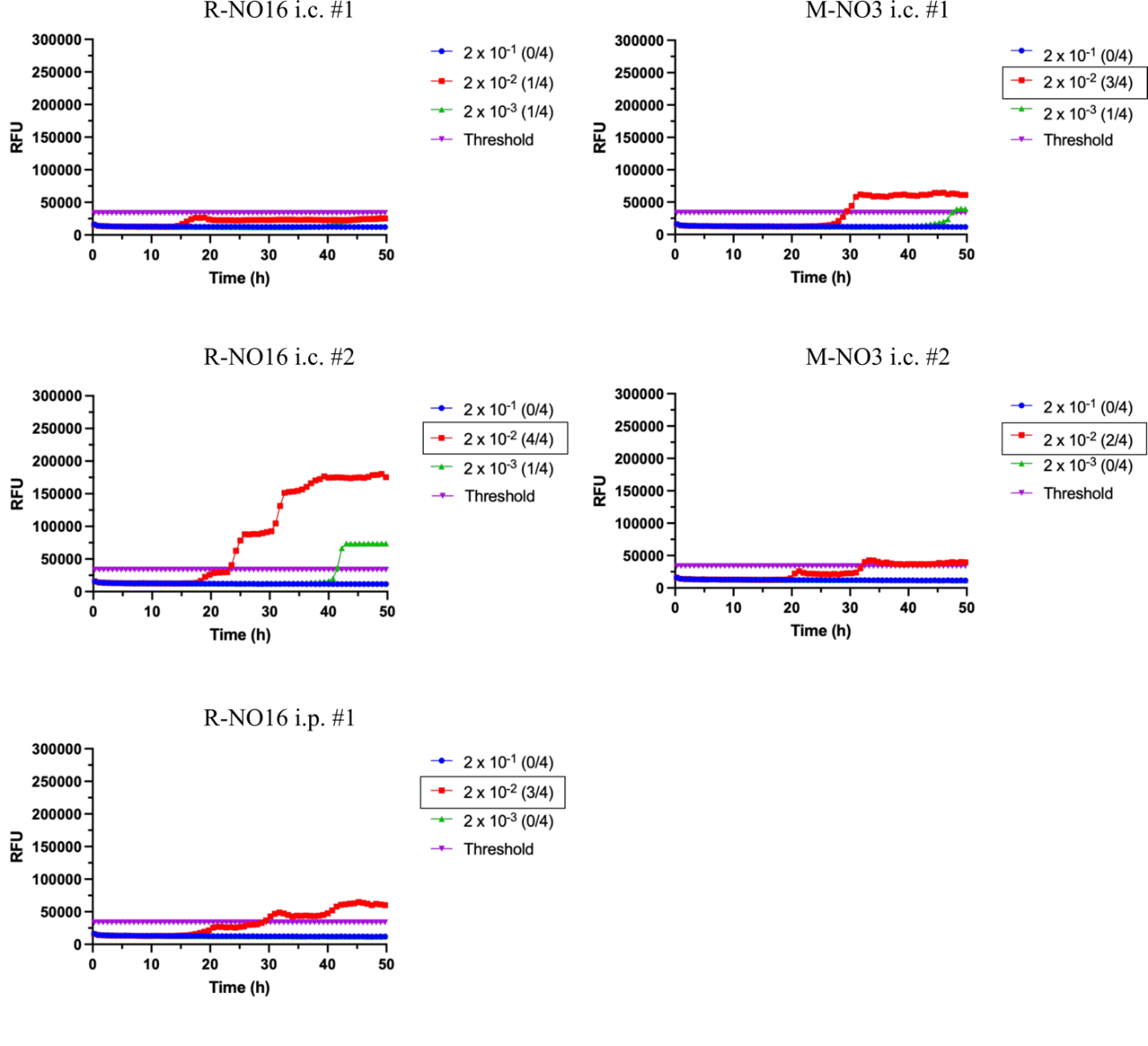


**Figure B.** **Seeding activity in PrP^res^-negative brains of *Prnp*.Cer.Wt mice inoculated with R-NO16 and M-NO3.** Left column shows RT-QuIC results from individual *Prnp*.Cer.Wt mice inoculated i.c. (n = 2) and i.p. (n = 1) with R-NO16 that were negative for PrP^res^ on western blotting (**Figure 2**). Right column shows RT-QuIC results from individual *Prnp*.Cer.Wt mice inoculated i.c. with M-NO3 (n = 2) that were negative for PrP^res^ on western blotting (**Figure 2**). Samples were considered positive when a minimum of two out of four reactions crossed the threshold relative fluorescence unit (RFU), indicated by the violet line. Positive dilutions are highlighted in black rectangles. The threshold is the average RFU of all negative control reactions plus five times their standard deviation. Negative control was a non-inoculated *Prnp*.Cer.Wt mouse brain. The y-axis represents the RFU, and the x-axis represents time in hours (h).


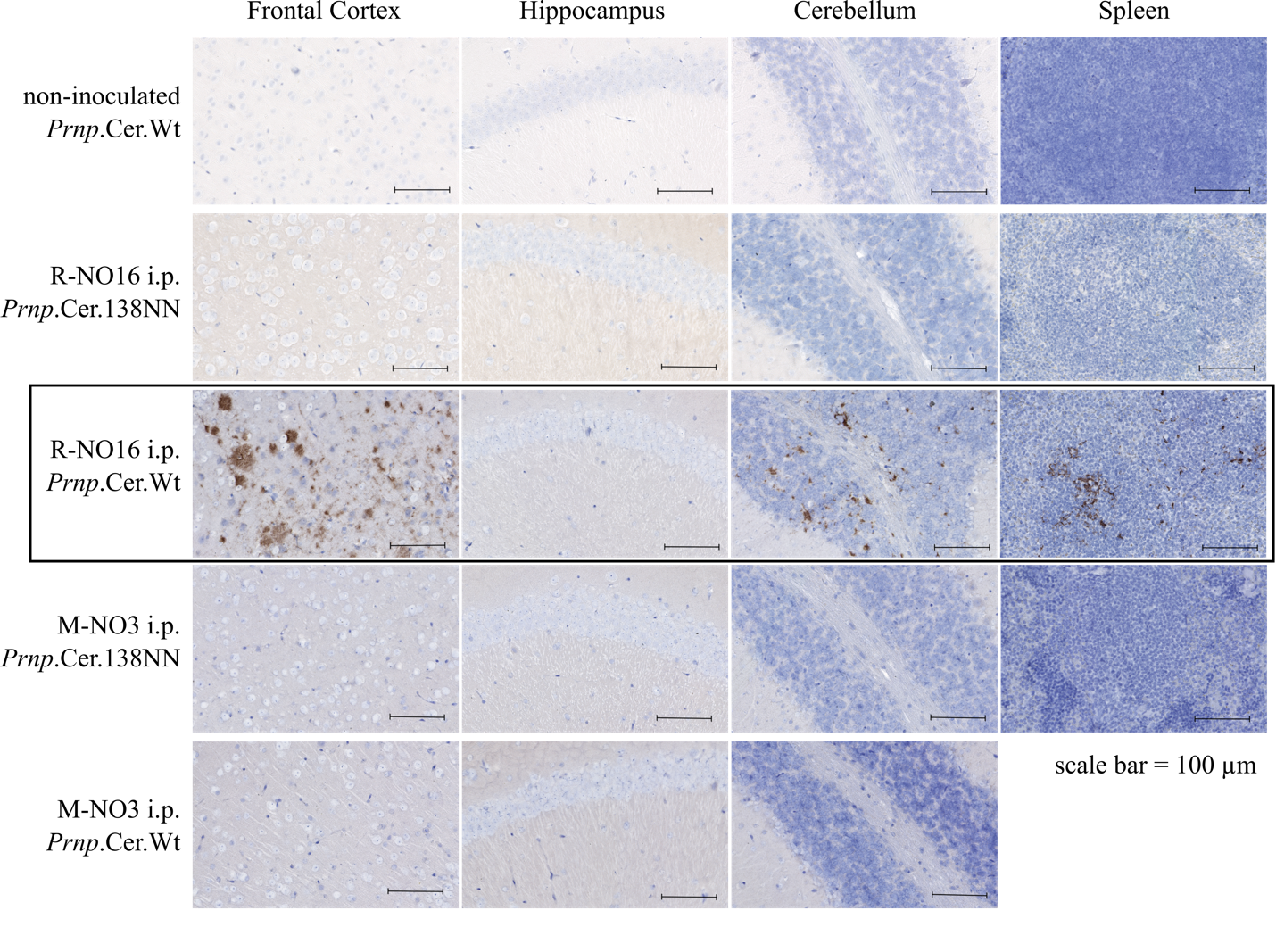


**Figure C. Representative immunohistochemistry of brain and spleen sections from gene-targeted mice inoculated i.p. with the R-NO16 and M-NO3 isolates.** Abnormal PrP deposits were detected in the frontal cortex, cerebellum, and spleen of the *Prnp*.Cer.Wt mouse inoculated with R-NO16 (row 3, highlighted with the black rectangle), but not M-NO3 (row 5). No abnormal PrP deposits were detected in *Prnp*.Cer.138NN mice tissues inoculated with both isolates (rows 2 and 4). No abnormal PrP deposits were detected in the hippocampus of all mice tested. Detection of abnormal PrP deposits was performed using the anti-PrP antibody BAR224 (1:2000) with hematoxylin counterstain.

**
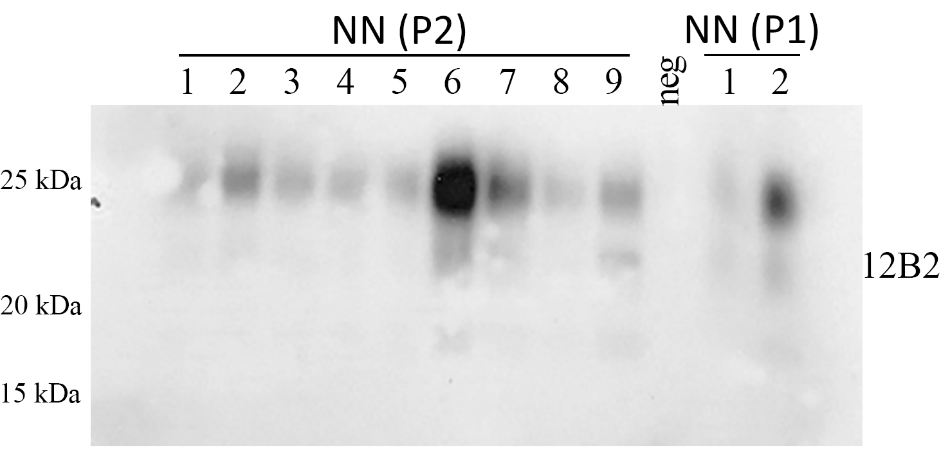
**

**Figure D. Western blot analysis of *Prnp*.Cer.138NN brain homogenates inoculated with M-NO3.** Brain homogenates of M-NO3 1^st^ and 2^nd^ passage were digested with 50 μg/ml of PK and subjected to western blot analysis using anti-PrP antibody 12B2. Non-infected *Prnp*.Cer.138NN brain homogenate served as a negative control.

**
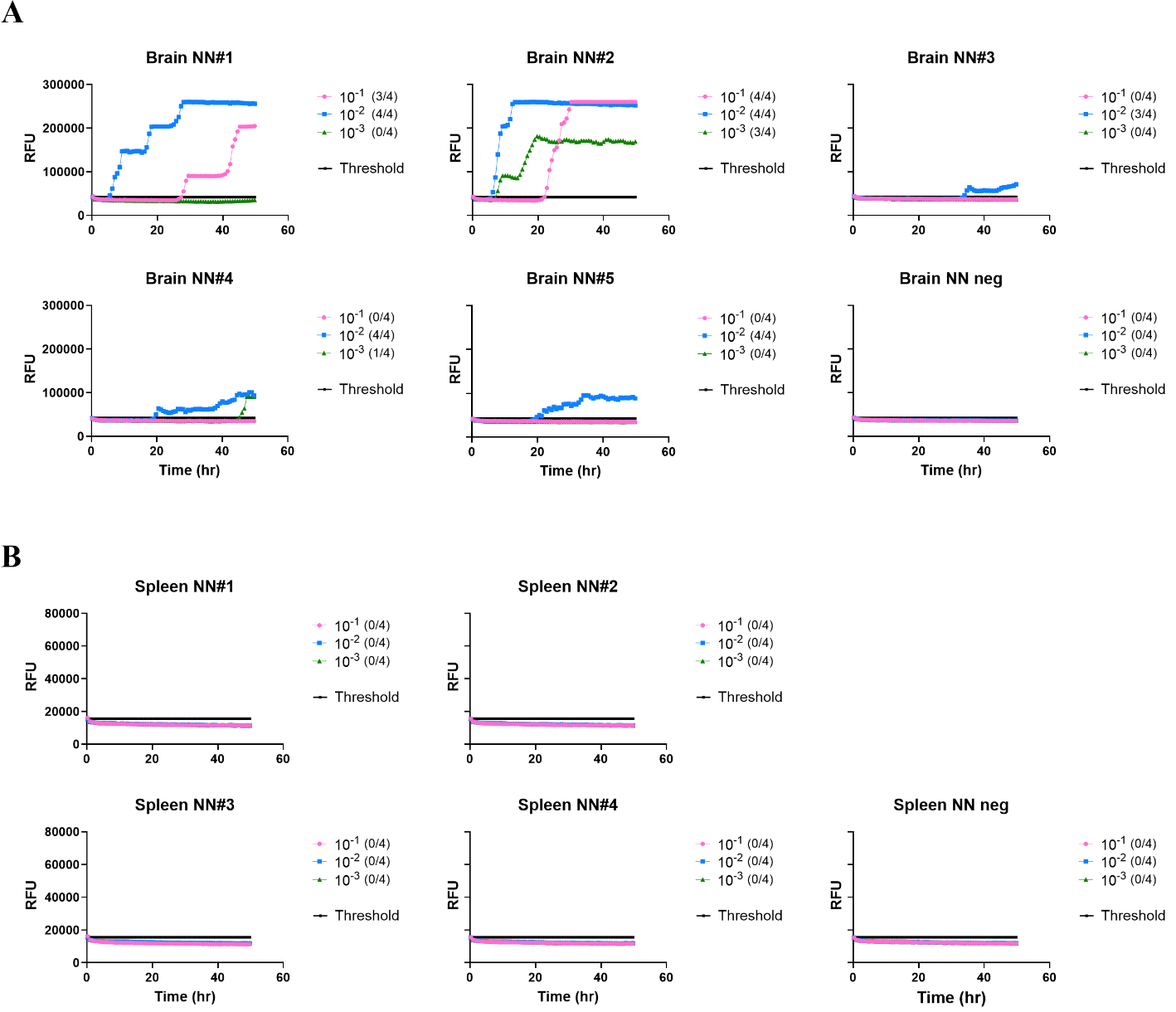
**

**Figure E. RT-QuIC analysis of PrP^res^-negative brains and spleens of *Prnp*.Cer.138NN mice inoculated i.p. with M-NO3. (A)** Brain (n = 5) and (B) spleen (n = 4) homogenates of *Prnp*.Cer.138NN mice inoculated i.p. with M-NO3, negative for PrP^res^ in western blot (**Figure 2**), were serially diluted and analyzed by RT-QuIC using mouse recombinant PrP as a substrate. Samples were considered positive when a minimum of two out of four reactions crossed the threshold relative fluorescence unit (RFU), indicated by the black line. The threshold is the average RFU of all negative control reactions plus five times their standard deviation. Negative control was non-inoculated *Prnp*.Cer.138NN mouse brain and spleen, respectively. The y-axis represents the RFU, and the x-axis represents time in hours (h).

**
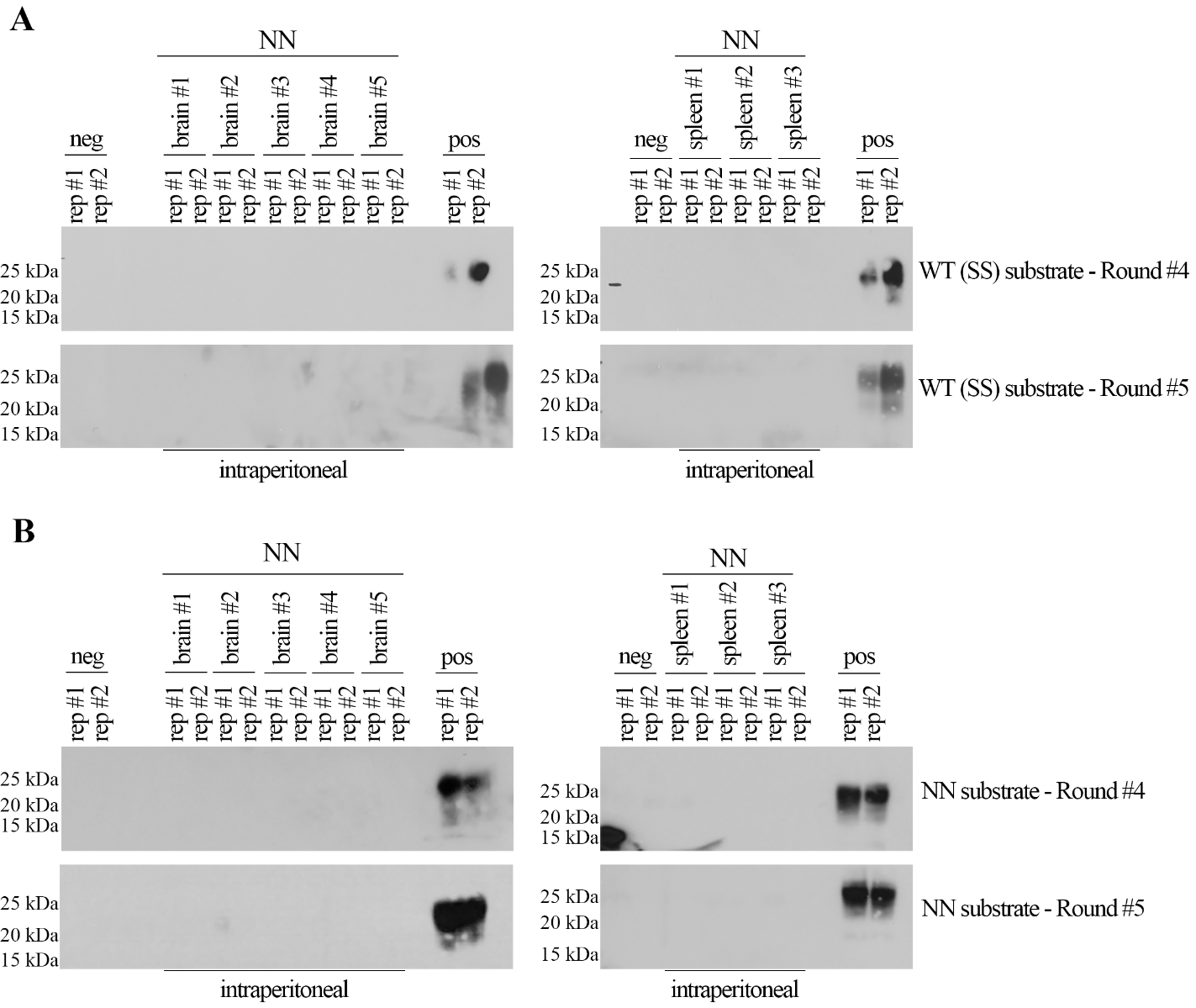
**

**Figure F. Serial PMCA of brain and spleen homogenates of *Prnp.*Cer.138NN mice inoculated i.p. with M-NO3.** 2 x 10-2 dilutions of brain (n = 5) and spleen (n = 3) homogenates of *Prnp*.Cer.138NN mice inoculated with M-NO3 were subjected to 5 rounds of PMCA using

naïve brain homogenates of either *Prnp*.Cer.Wt (**A**) or *Prnp*.Cer.138NN (**B**) mice as a substrate. sPMCA products of rounds 4 and 5 were digested with PK (50 μg/ml) for 1 hour and analyzed by western blot using anti-PrP mAb 4H11 (1:500). Naïve brain homogenate was used as a negative control, brain homogenate of *Prnp*.Cer.Wt mice inoculated with R-CA1 served as a positive control.

**
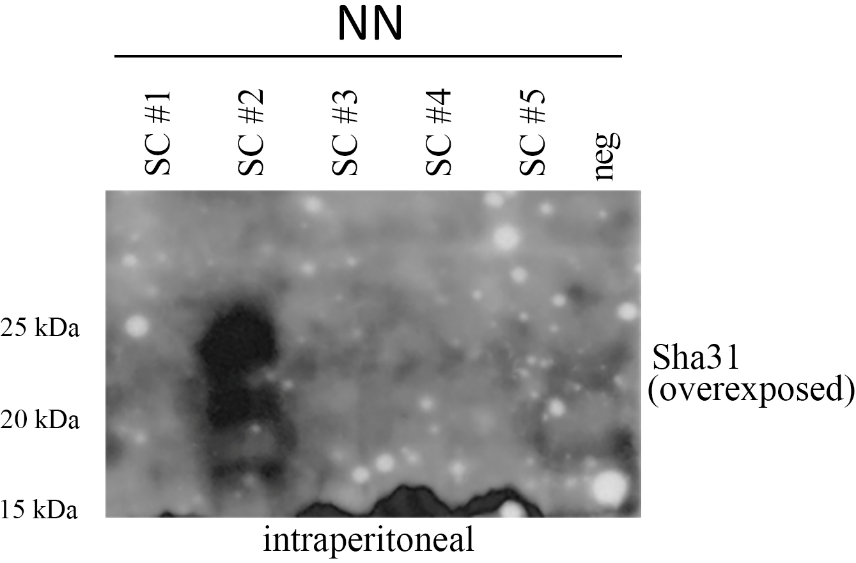
**

**Figure G. Western blot analysis of spinal cord homogenates.** Spinal cord homogenates of *Prnp*.Cer.138NN mice inoculated i.p. with M-NO3 were digested with 50 μg/ml of PK and analyzed by western blot using anti-PrP antibody Sha31 (1:10,000). This is an overexposed version of the western blot shown in **Figure 4B (lower panel)**.
