# Supplementary material for: Norwegian moose CWD induces clinical disease and neuroinvasion in gene-targeted mice expressing cervid S138N prion protein
Source: PLoS Pathog. 2024 Jul 1;20(7):e1012350. doi: 10.1371/journal.ppat.1012350 (PMC11244775; doi:10.1371/journal.ppat.1012350)
Supplement: Attachment — Submitted filename: Arifin Hannaoui et al PLoS Pathogens_response.pdf [file ppat.1012350.s003.pdf]

Response to Reviewers *PPATHOGENS-D-23-01939 R1*

First of all, we would like to thank the referees for their time to assess our manuscript. We are grateful for the constructive feedback, which we were able to incorporate in a substantially revised version of our manuscript. Please find a detailed response to each comment below. Note that line numbers indicated below refer to the manuscript version without tracked changes.

Part II – Major Issues: Key Experiments Required for Acceptance

Reviewer #1: Title and elsewhere: A claim is made that Norwegian moose CWD gets to the brain independent of the spleen, but no timepoint samples are analyzed and the potential for some degree of PrP Sc degradation is not addressed. This other work should be done or these claims should be softened and these other caveats should be explained somewhere in the manuscript.

Response: We have removed 'spleen-independent' from the title and changed wording to include alternative explanations in the abstract (lines 46 – 48), author summary (lines 61 – 64) and introduction (lines 105 – 106). We also added a paragraph to the discussion about alternative explanations for the lack of prion seeding activity in spleens, and its potential impact on neuroinvasion (lines 301 – 318).

Reviewer #2:

1. The current study is incremental, as it provides additional information on isolates that have been described in other publications.

Response: We hope that with the revised version of our manuscript we were able to convince the reviewer of the novelty of our findings. Even though one out of the three isolates we used (R-NO16 and H-NO1, as mentioned in the manuscript, were not included in previous publications) has been characterized before with regards to strain properties, the main goal of our study was to determine transmissibility to a model expressing a PrP variant that has been associated with 'genetic resistance', and to address whether especially the moose and red deer isolates that were shown not to be lymphotropic are capable of neuroinvasion upon peripheral/intraperitoneal infection. Both of these questions have not been addressed before, and notably, in our revised manuscript, we were able to strengthen the evidence of neuroinvasion of the moose isolate, as detailed below.

2. Detection of prions in brain and spleen of non-clinical animals is provided by RT-QuIC. To provide more convincing evidence of subclinical infection, these samples should also be tested in a secondary method (e.g., PMCA, cell assays, bioassay).

Response: We have decided to re-analyse the spleen and brain homogenate samples by serial PMCA as we have not established CWD cell assay in the lab, and bioassays would take close to 2 years. We used a $2x10^{-2}$ seed dilution and either Prnp.Cer.Wt or Prnp.Cer.138NN brain homogenates as substrate and performed 5 rounds of serial PMCA. Different PMCA conditions were tested, with variable sonication and amplification times and sonicator output, which resulted in consistent amplification of the positive control. We were not able to amplify seeds from the spleen and brain homogenates under any of these conditions, and a representative result

is shown in a **new S6 Figure.** It is known that RT-QuIC and PMCA do not have identical sensitivities, which we have experienced previously when testing CWD field samples by RT-QuIC that were tested in parallel by our collaborator's group with PMCA. Several samples were found to be positive in RT-QuIC but negative in PMCA, and with follow-up bioassays we were able to confirm prion infectivity in such samples. Therefore, we conclude that under the experimental conditions used here, RT-QuIC is more sensitive than sPMCA. However, the most surprising results in the clinically negative mice was that we detected seeding activity in the brains of Prnp.Cer.138NN mice inoulated i.p. with M-NO3. Therefore, we focused on supporting the conclusion that M-NO3 is capable of neuroinvasion. We analysed spinal cord homogenates by RT-QuIC and western blot, and were able to detect seeding activity as well as PrPres in the spinal cord of one mouse. These additional data are now included as **new Figure 5** and new **S7 Figure**. Furthermore, for brain and spleen homogenate samples of the Prnp.Cer.138NN group inoculated i.p. with M-NO3, we added the RT-QuIC graphs as **new S5 Figure** to support the results summarized in Table 2.

3. Western blot of the original inocula used for this study should be included. The PrPSc generated in mice upon inoculation of the European CWD strains should be compared, side by side, with this inocula and with other CWD strains already characterized.

Response: Thank you for this suggestion, we have added a **new Figure 2D** to compare PrPres patterns generated in the gene-targeted mice with a variety of CWD isolates.

4. The potential isolation of different strains is a relevant result. As such, this needs to be further characterized, including second passages in transgenic mice. Although it is understandable that these experiments take long time, they are needed to draw solid conclusions (as presented and discussed by the authors).

Response: We have added results from 2nd passage as **new Figure 4** and **new S4 Figure**. We observed a significantly shortened survival time and a full attack rate in the 2nd passage. Analysis of PrP^{res} by western blot using different antibodies revealed that mainly 12B2-positive PrP^{res} was retained from passage of a pooled brain homogenate. Since the main focus of our manuscript was not on full strain characterisation but on transmission barrier and neuroinvasion, we did not pursue experiments to further characterize biochemical or neuropathological differences, beyond detection by and presence of N-terminal epitopes using the 12B2 antibody as an indication of conformational differences.

Part III – Minor Issues: Editorial and Data Presentation Modifications

Reviewer #1:
Abstract- a couple of suggestions that may be difficult depending on word limits-
Line 32-33- What do you mean by subclinical infection? Evidence of PrPSc, but not clinical signs at time of euthanasia?

Response: We changed the wording in the abstract (lines 35 – 36) and defined the term in the introduction as suggested (lines 99 – 100).

Line 35: can you address the potential titer issue here? RT-QuIC suggests maybe we could have expected reindeer to incubate faster if amount only.

Response: We understand the concern, but we cannot comment on titer beyond what has been stated with regards to RT-QuIC results and seeding activity, as we did not titrate infectivity of the isolates. The main point though is that M-NO3 is a different strain of CWD, which we argue is the main reason for breaking the transmission barrier we observed previously in this mouse model when infected with a variety of CWD isolates (see Arifin et al., 2023).

Line 52: again, what is subclinical infection to you? This can be misleading if mice were euthanized prior to the time that would be required to develop clinical signs.

Response: We have now defined the term 'subclinical infection' with respect to our observations in the introduction. Another term to use might be 'pre-clinical', but this would imply that the animals develop disease, and therefore, it is also not entirely correct, and we can only speculate if and when the mice would develop disease if they had a longer natural lifespan. Therefore, we hope that we were able to clarify any concerns by adding a definition of 'subclinical' in the introduction.

Introduction- associated with paragraph startint at line 81-
Could you introduce what you are referring to for the various genotypes (wt, deer, caribou, elk) how they are the same/different and put the terminology here that will be used throughout because this isn't actually clearly explained until the methods, which occurs at the end.

Response: More clarification of the genotypes and respective species has been added in lines 95 – 97.

Line 86: which deer?

Response: See comment above.

Line 94: one potential place to address difference between spleen independent vs transient splenic replication.

Response: We have included a statement on potential transient spleen replication in lines 105 – 107.

Line 100: please add the substrate used here. It is not in the M&M and only appears in the figure legend.

Response: It has been included, in addition to the description in the materials and methods section.

Line 106-107: Is it an expectation that all of these will replicate equally in the substrate and the differences are due to amount of PrPSc present?

Response: Yes, this was an expectation, and in our experience, we have been able to amplify a number of different CWD isolates successfully with the mouse recombinant PrP.

Line 118: Thoughts on the higher attack rate after IP inoculation (maybe a comment in the discussion)?

Response: We added a comment in the discussion, lines 335 – 339.

Line 120: Please comment on the incubation period allowed for the wt mice with the M-NO3 inoculum. This does not seem long enough ensure that all of these were negative since the IC incubation period was 700 +/- 108 days.

Response: We added additional explanation in the results section, lines 133 – 136 and in the materials and methods section, in addition to the details provided for the different endpoints in the footnotes of Table 1. Experimental endpoints were selected according to the natural lifespan of mouse models and to be comparable between groups of mice with different genotypes inoculated with the same CWD isolate, e.g. *Prnp*.Cer.wt and *Prnp*.Cer.138NN mice inoculated i.p. with M-NO3 (**Table 1**).

Line 129: (doesn't require a response) Western blot may not be sensitive to identify cases that would be detectable by other methods (ELISA, for example). It takes a relatively high OD/strong positive by ELISA before WBs are positive without some enrichment method. It would be better to call these non-detect rather than negative.

Response: We completely agree about the differential sensitivities of western blot, ELISA, and other detection methods.

Line 137: ... activity in either their brains or spleens

Response: This has been changed.

Line 151: suggest omitting strong

Response: We omitted strong.

Line 160: omit was able to break this transmission barrier and

Response: We opted to not omit this statement as it reflects that the moose isolate was able to break the transmission barrier, i.e. produce clinical disease in this specific mouse model that did not develop clinical disease when inoculated with any other CWD isolate we tested.

Line 179: please add something to the title of this section, so it is clear that you're talking about WB's

Response: We added in line 204 that samples were analyzed by western blot.

Line 209: Is 258 days enough to make a claim? This isolate took 700 days in the other mouse strain.

Response: We stated that none of the TgElk mice developed disease up to 258 dpi when inoculated with M-NO3, which is correct. Unfortunately, the specific transgenic model used here (TgElk) starts to develop non-prion related health issues at around 300 days, and with this, we have to choose earlier experimental endpoints.

Line 212-213: Worth looking at Angers, Science, 2010 and considering a reference as this codon has been identified with strain-associated replication differences.

Response: This has been added to the discussion, lines 356 – 358.

Line starting 216: is there a way to add something about the inoculum (species of origin) from the previous studies, so the reader knows how to categorize the information?

Response: We are not quite sure what is required here. We included all available information in the materials and methods section and introduction, including citations of previous publications describing the isolates. We have added more clarification with regards to genotypes and species, and hope this clarifies the concern.

Line 237: no clinical disease at what DPI?

Response: This has been added, line 295 (up to 750 dpi).

Line 239: again there is the potential that at some point clean up outpaces accumulation

Response: We have added more discussion including references to previous work discussing the balance between degradation and replication of prions (lines 301 – 318).

Line 245: What about hematogenous spread?

Response: We mentioned one minor pathway of neuroinvastion that we think is relevant in our study, and did not discuss hematogenous spread as there is currently no evidence suggesting that prions can be absorbed into blood vessels in the peritoneal cavity.

Line 271: please clarify this sentence- you can transmit CWD prions (but not clinical disease)

Response: We have changed ‘transmit’ to ‘induce’ in the sentence in line 350.

Line 286: as above, is without involvement of the spleen is not the same as not being able to detect PrPSc at the endpoint.

Response: We have softened the language in lines 368 – 370 to include the possibility of transient replication in the spleen.

Line 288: strains and genotypes that define. . .

Response: This has been edited as suggested.

Line 299: please describe this genotype in the same way as for the mouse strains. This is hard to follow.

Response: Additional clarification has been added in lines 383 – 390. For the genotype of the specific reindeer isolate, we have used the nomenclature suggested by the authors of the original publication cited (61) for consistency.

Figure 4: how would these migrations compare to the reindeer and moose isolates in figure 3?

Response: We did not include the brain homogenates from H-NO1 inoculated TgElk mice for comparison since the results shown in former Figure 4 (now Figure 6) were from gene-targeted mice, and the different PrP^C expression level is a variable that might affect the outcome of PrP^{res} patterns.

Reviewer #2:

1. M-NO3 is mentioned in the abstract. Specific information should be avoided in this section, as it is confusing at this point. A more general message, and no specific data (on a specific isolate) should be mentioned in this section.

Response: We have removed all specific information from the abstract and the author summary.

2. In Table 1, Elk should be listed in the last column to agree with the flow of the manuscript.

Response: We have edited Table 1 as suggested.

3. Table 1. It is not clear why the animals noted with "b" are included in the table.

Response: We have included those endpoints for full transparency about the fate of every single mouse in the experiments, including animals that were euthanized at the overall endpoint when the experiment was terminated without signs of clinical disease, i.e. "b". It is supposed to provide better 'visualization' of the numbers for readers, when comparing between isolates and how long the mice were kept alive for.

Reviewer #3: I have no minor issues.

Response: Thank you very much!
